# In Vivo Cone Photoreceptor Topography of the Human Foveola

**DOI:** 10.1167/iovs.66.11.13

**Published:** 2025-08-06

**Authors:** Julius Ameln, Jenny L. Witten, Aleksandr Gutnikov, Veronika Lukyanova, Frank G. Holz, Wolf M. Harmening

**Affiliations:** 1 Department of Ophthalmology, University of Bonn, Bonn, Germany

**Keywords:** photoreceptors, adaptive optics, cone density, foveal topography, AOSLO

## Abstract

**Purpose:**

To study in vivo cone topography of the normal human foveola.

**Methods:**

The fovea in both eyes of 30 healthy participants was imaged with adaptive optics scanning light ophthalmoscopy. High-resolution image montages spanning two degrees of visual angle were created and cone center locations annotated. Continuous cone density maps were computed by a Voronoi cell area approach to also yield the topographical center, the cone density centroid (CDC). Cone density profiles were extracted and fit with a four-parameter decay function, D = D_0_ / (1 + (E/*a*)*^b^*)*^c^*, with D as cone density (cones/mm^2^), D_0_ as cone density at the CDC, and E as eccentricity (µm).

**Results:**

Across eyes, D_0_ was 175,474 ± 20,543 cones/mm^2^, on average (range 136,001–216,209 cones/mm^2^). Density dropped anisotropically along the meridians, shallower horizontally, with average best fit parameters (*a, b, c*) of 61.95, 2.469, 0.268 for horizontal, and 59.11, 2.012, 0.357, for vertical profiles, respectively. In radially averaged profiles, cone density reached 50% of D_0_ at 151 ± 17 µm eccentricity (range 128–193 µm). Temporal cone density was slightly higher than nasal. Most topographical metrics were highly correlated between fellow eyes.

**Conclusions:**

Despite a 1.6-fold range in absolute cone density, foveolar density profiles could be well described by a sigmoidal decay function across all eyes. This established a normative cone density profile of the healthy foveola. It allowed cone density estimation in cases of only partially available data, which alleviates resolution demands for future studies and renders possible retrospective analyses of foveolar cone topography in sub-optimal imagery.

The foveola, the central 1° diameter of the retina, plays an outstanding role in human vision. Here, only cone photoreceptors are present, while second and third order neurons are displaced centrifugally, promoting undisturbed light capture.^1^^–^^3^ The light-sensitive cone outer segments are maximally thinned and elongated in the foveola, creating higher packing densities than anywhere else in the retina.^4^ Together with a spectrally diverse opsin outfit and a post-receptor circuitry that maintains spatial granularity, it is the arrangement of the light detectors of the foveola that enables us to see in fine and colorful detail.^5^^,^^6^

Studying photoreceptor topography in the human foveola (i.e., cell density, arrangement, and their associated variability) is of increasing interest to ophthalmology, vision science, and the neurosciences.^7^^–^^11^ At the same time, topographical analysis of this region of the retina has proven to be exceptionally challenging, a consequence of its particular morphology. In histological preparations, the foveola's delicate structure is difficult to preserve because minimal mechanical forces can disrupt its order.^12^^,^^13^ With the limited availability of unblemished donor tissue, topographical analysis of the normal foveola based on histology is prone to high variability.^4^^,^^12^^,^^14^ In comparison, in vivo retinal imaging is challenged with the limitations set by the optical properties of the eye, where the outer segments of foveolar cones are—because of their minimal diameter—on the brink of resolvability. With high-resolution ophthalmoscopy using adaptive optics, the aberrations of the eye can be compensated and foveolar cones become resolvable in the living eye.^15^^–^^17^ This has sparked a number of studies using adaptive optics scanning light ophthalmoscope (AOSLO) imaging, where the cellular topography was directly or indirectly quantified^18^^–^^20^ in relation to visual function,^21^^–^^23^ eye development,^10^^,^^23^ and retinal disease.^7^^,^^24^^–^^26^ Common limitations regarding topographical analyses in these studies are relatively small and discontinuous areas of analysis, only partially annotated regions of interest, or a low number of examined eyes (Table 1). As a consequence, adaptive optics imaging studies describing the healthy state of the foveola's photoreceptor mosaic rely on a limited number of datasets.

**Table 1.** Literature on Human Foveal Cone Density

Reference	No. of Participants (No. of Eyes)	Central Cone Density (Cones/mm^2^ × 1000), Range or Mean ± SD	Approximate Foveal Analysis Area (Central Area Diameter; Highest Eccentricity)	Cone Annotation and Density Calculation	Comments
Østerberg^3^	1 (1)	147.4	Horizontal meridian	NA, square: 20 × 20 µm	Histology
Hartridge^63^	1 (1)	127.0	NA	NA, rectangle: 67 × 58 µm	Histology
O'Brien^64^	2 (2)	218.3 and 288.6	Horizontal meridian	NA	Histology
Miller^65^	1 (1)	128	Horizontal meridian	NA	Histology
Farber et al.^66^	1 (1)	49.6	Horizontal meridian	NA	Histology
Yuodelis and Hendrickson^4^	2 (2)	120 and 208.2	Horizontal meridian	NA	Histology
Ahnelt et al.^14^	2 (2)	178 and 238	Horizontal meridian	NA, square: 50 × 50 µm	Histology
Curcio et al.^12^	7 (8)	98.2–324.1	20 mm	Discrete ROI	Histology
				Rectangle: 43 × 29 to 130 × 88 µm	
Begin of in-vivo imaging					
Carroll et al.^16^	1 (1)	148.8	1°	Full annotation Circle: 20.6 µm radius	
Putnam et al.^17^	3 (3)	148.8–226.9	1°–2.5°	Full annotation Circle: 20.6 µm radius	Data subset presented in reference 16
Li et al.^30^	4 (4)	123.8–167.7	2°	Full annotation Window: 150 cones	
Merino et al.^67^	3 (3)	128.3–147.3	0.7°–1.2°; at multiple locations up to 10°	Discrete ROI Inter cone distance	Cone density assuming hexagonal cell mosaic
Wilk et al.^68^	9 (9)	84.7–165.1	0°–20° temporal	Discrete ROI Square: 25 × 25 to 45 × 45 µm	
Zhang et al.^18^	20 (40)	136.1–247.1	10°	Discrete ROI	
				Square: 5 × 5 to 40 × 40 µm	
Cooper et al.^34^	20 (20)	119 ± 23.3	11° meridional	Discrete ROI	
				Square: 37 × 37 to 100 × 100 µm	
Wells-Gray^69^	5 (5)	164 ± 24	0°–30° horizontal meridian	Discrete ROI Square: 35 × 35 to 60 × 60 µm	
Wilk et al.^70^	22 (22)	106.7–214	FAZ area	Discrete ROI Square: 25 × 25 to 45 × 45 µm	Data subset presented in references 34, 68, and 71
Wilk et al.^71^	23 (23)	106.7–214	FAZ area	Discrete ROI Square: 25 × 25 to 45 × 45 µm	Data subset presented in references 34, 68, and 70
Wang et al.^10^	16 (28)	118.5–204	1.7°	Full annotation Circular: 5 arcmin radius	
Cava et al.^20^	58 (94)	122.1–247.7	1°	Full annotation Square: 150 cones	Data subset presented in references 70 and 71
Domdei et al.^72^	4 (4)	13.7–18.4 [cones/deg²]	1°	Full annotation 150 closest cones	
Reiniger et al.^23^	21 (41)	145.9–221.9	1°	Full annotation 150 closest cones	Data also presented in reference 22
Wynne et al.^19^	44 (44)	117.6–220	1°	Full annotation	Data subset presented
				NA	in reference 20
Baraas et al.^36^	12 (12)	105–163.8	2° square; 6° horizontal; 3° vertical	Discrete ROISquare: 10 × 10 to 50 × 50 µm; circle: 25 µm radius; including 150 cones	
Domdei et al.^73^	10 (20)	147–215.7	2°	Full annotation 150 closest cones	
Heitkotter et al.^74^	42	125.5–249.3	1°	Full annotation Square: including 150 cones	Data subset presented in^20^^,^^70^^,^^71^
Warr et al.^47^	44 (44)	117.9–258.3	1°	Full annotation Square: Different #cones	Data subset presented in references 19, 20, 70, and 71
Adhan et al.^43^	19 (19)	189 ± 21.7 146–233	1.7°	Full annotation Square: 150 cones	Data subset presented in references 19, 20, 70, and 71
Wang et al.^37^	11 (17)	∼6.3–11.8 [cones/deg²]	10°	Discrete ROI	Data extracted from
				NA	figure
This study	30 (60)	129.8–216.2 175.6 ± 21	2°	Full annotation 150 closest cones	Data subset partially presented in references 23 and 73

FAZ, foveal avascular zone; ROI, region of interest.

To meet the need for a comprehensive topographical description of the foveola, three features are desired: (1) An analyzed area of about 2° in diameter to include the full two-dimensional extent of the central density elevation, (2) a continuously and carefully annotated cone mosaic to allow a dense sampling of the rapidly changing cone topography, and (3) a large enough case number to adequately represent the typical variance present in human foveolar cone mosaics. Recent improvements in AOSLO image analysis pipelines such as automated montaging^27^ and neural network assisted cell annotation^28^^,^^29^ enable analysis of larger continuous areas of the human cone mosaic in a timely manner by reducing the human workload in the process. Here, we use such approaches and pair them with manual corrections in each step for highest data fidelity.

We present the human photoreceptor topography in a continuously annotated ∼2° diameter of the foveolar center. We further studied the potential of estimating cone density profiles in eyes where the smallest foveal cones are unresolved—for example, because of limited instrument resolution or in retrospective data analysis. Our normative data set and the derived profile functions can be used in clinical studies evaluating foveal photoreceptor topography.

## Methods

### Human Participants

A total of 30 participants (60 eyes) with fully resolved foveal centers were included in the study. Participant age ranged from 10 to 44 years, with a mean age of 25.2 ± 7 years. Twenty-three participants were female. All participants were screened by an ophthalmic expert prior to participation to ensure retinal health and eligibility of participation. Optical swept-source biometry (IOL Master 700; Carl Zeiss Meditec, Dublin, CA, USA) was performed to determine the eye's retinal magnification factor based on a measurement of axial length, corneal radii and anterior chamber depth.^30^ Mydriasis and cycloplegia were induced by instilling two drops of 0.5% tropicamide 15 minutes before an imaging session, with possible re-drops if necessary. All participants gave informed written consent, with minors co-signing alongside their custodians prior to enrollment. The study adhered to the tenets of the Declaration of Helsinki and was approved by the independent ethics committee of the University of Bonn (no. 009/13, approval: October 16, 2023).

### Foveal Imaging

**Figure 1.**
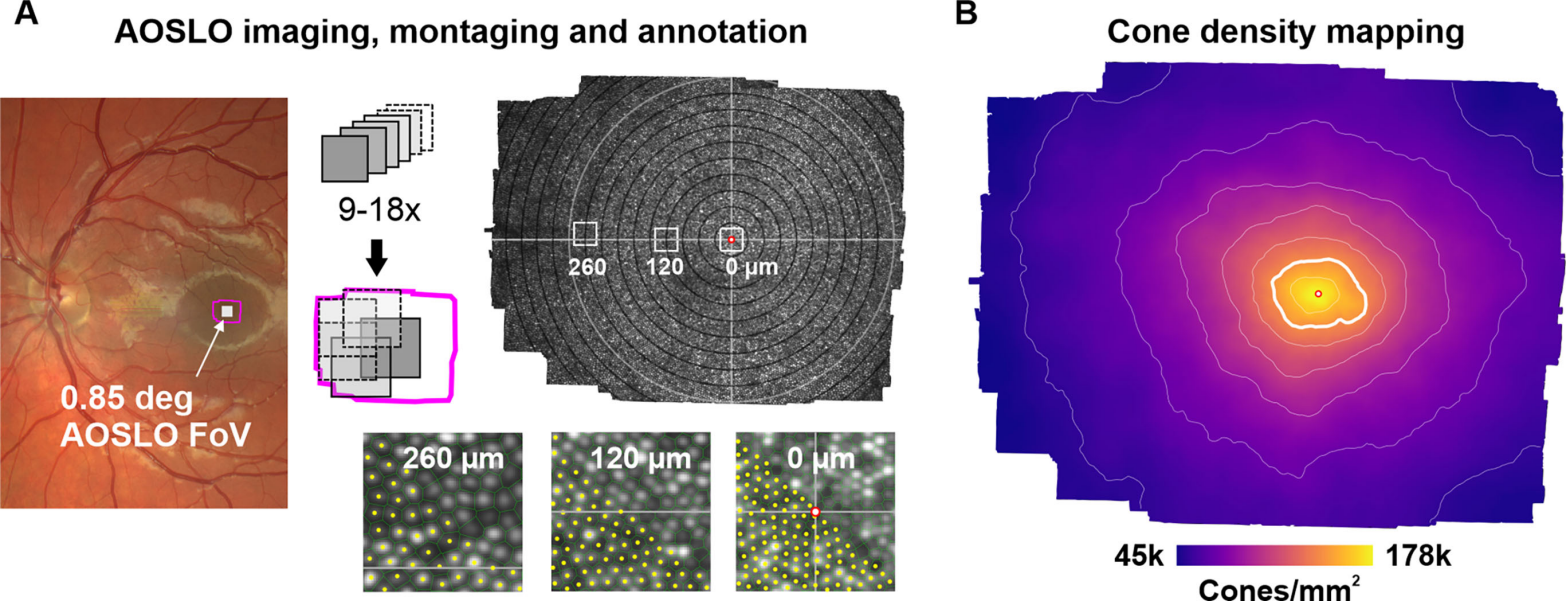
Cone density mapping from AOSLO images. **(A)** Fundus image with foveal AOSLO montage location (*magenta outline*) and AOSLO field of view (*white square*). Nine to eighteen overlapping AOSLO videos were used to create a continuous montage of the foveal center. *White circle* in the montage is the 2° diameter. *White outlines* at 0, 120, and 260 µm eccentricity are magnified below and show exemplary cone center annotations. **(B)** Density map computed from fully annotated montages. Color is cone density. Contour lines indicate 10% iso-density contour steps with the *thick white line* representing the top 20% density contour. A *red-white circle* marks the location of the cone density centroid (CDC), representing the topographical center of the density map and center of the foveola.

For each participant, a personalized dental imprint (bite-bar) was made to position and control the head in front of the imaging system. A modified version of a previously described custom-build confocal AOSLO was used to record retinal videos. The instrument comprised a broadband laser source (SuperK EXTREME EXR-20; NKT Photonics, Birkerod, Denmark) to provide different light channels with center wavelengths of 711 (±12), 788 (±9.5) or 840 (±6) nm, by serial bandpass and dichroic filtering (FF01-711/225-25, FF01-788/12-25, FF01-840/12-25; Semrock, West Henrietta, NY, USA). A Shack-Hartmann wavefront sensor (SHSCam AR-S-150-GE; Optocraft GmbH, Erlangen, Germany) and a deformable mirror (DM97-08; ALPAO, Montbonnot-Saint-Martin, France) ran in closed loop using custom software to compensate ocular aberrations in real time. Wavefront sensing was performed using the respective imaging wavelength. An acousto-optic modulator (AOM, TEM-250-50-10-2FP; Brimrose Corporation of America, Sparks Glencoe, MD, USA) provided fast light switching to encode a movable fixation target into the imaging raster.^31^ A sub-Airy disc diameter pinhole (20 µm) was placed in front of the photomultiplier tube (H4711-50; Hamamatsu Photonics, Hamamatsu, Japan), whose output was sampled by a field programmable gate array (Vitrex-5 FPGA ML506; XILINX, San Jose, CA, USA) to produce video frames of 512 × 512 pixels at a frame rate of 27 or 30 Hz. Imaging raster size was 0.85°; thus the digital spatial sampling rate was 600 pixels per degree of visual angle (6 arcseconds per pixel). Raster desinusoiding was achieved by a static pixel re-assignment determined with spatial calibration of a low-reflectance grid target (62–949; Edmund Optics Inc., Barrington, NJ, USA). Multiple five- to 10-second long videos were recorded while participants were instructed to look at either a small, 1.6-arcmin fixation target presented within the imaging field (3Hz flash, 50% duty cycle) or at eight evenly spaced points formed by the raster corners and borders. Images were thus centered on the preferred retinal locus of fixation and eight surrounding locations, forming an overlapping imaging field that covered about 2° of the foveal center (Fig. 1A). This imaging pattern guaranteed to capture the cone density peak of the foveola, given that the offset between the cone density centroid (CDC) and preferred retinal locus of fixation was found to be small in the healthy retina, less than 10 arcmin on average.^17^^,^^23^ To maximize success rate of resolving all foveal photoreceptors, videos were recorded at various defocus settings of the deformable mirror (±0.02 Diopters) and later processed as described in the next section. The total imaging time, from first to last AOSLO video recording, was on average 30–45 minutes per participant.

### Image Processing and Annotation

Single AOSLO videos were stabilized offline using a modified version of a strip-wise image registration algorithm^32^ to yield high signal-to-noise images of each retinal location. Modifications to the registration algorithm entailed improved intensity-based “bad” frame removal that excludes edge cases, optimal seed frame selection via cross correlation across all frames, and removal of any frame parts containing residual distortions based on uncorrected microsaccades. Single summed and normalized images were then automatically aligned using software previously described,^27^ and were blended manually by prioritizing image quality in Corel Photo-Paint (CorelDRAW Graphics Suite 2019; Alludo, Ottawa, Canada). This created a seamless montage that minimized small residual image distortions (Fig. 1A). A custom MATLAB software, ConeMapper, was used to semi-automatically annotate cone center locations in the montages.^33^ Manual annotation corrections, performed by a single image grader only (author JA), assumed near hexagonal cone arrangement in cases of false-negative or false-positive results in the output of the automatic detection. To assess grading quality, a subset of the data (35 eyes) was independently manually corrected by another grader (author JLW) and compared (see Supplementary Fig. S6).

### Cone Density Profiles and Data Fitting

For each eye, two-dimensional maps of cone density were computed and further analyzed (Fig. 1B). In such maps, cone density was computed for each image pixel by first finding the 150 nearest cone centers given by their Euclidian distance to that pixel and then dividing the total area of the Voronoi tessellated cells by 150.^23^ The peak cone density (PCD) was the single highest density value within the map. The CDC, a singular retinal location, was defined as the weighted centroid in the contour area enclosing the top 20% of cone density values and is calculated using the *regionprops* function in MATLAB. We defined the topographical center of the fovea (eccentricity = 0) to be at the CDC. The cone density value at the CDC is referred to as D₀ throughout the article. All angular retinal units were transformed into linear units using the AOSLOs image magnification factor (600 pixels/degree) and the individual retinal magnification factor of each eye, allowing data comparison in linear space.

For each eye, density was extracted along the horizontal and vertical meridian passing through the CDC within isosceles triangle sectors with a vertex angle of 5° at the CDC and reported as the average value per eccentricity in single pixel steps (0.1 arcmin/pixel). Similarly, radially averaged density profiles were computed as averages of all density values falling onto a circle with eccentricity as radius and the CDC as center. Normalized density profiles were computed by scaling local density by D_0_. Average intercone distance profiles were smoothed by a moving mean of 11 µm. Horizontal, vertical, and radial average cone density profiles were fit to a four-parameter sigmoid function of the form:

(1) $$\begin{array}{c} D\left(E\right)=\frac{D_{0}}{{\left[1+{\left(\frac{E}{a}\right)}^{b}\right]}^{c}} \end{array}$$

with D being cone density (cones/mm^2^) at eccentricity E (µm), and D_0_ being the cone density at the CDC. Fit parameters, {a, b, c}, were found by the Matlab function “fit” (Method: Nonlinear least squares; robustness: bisquare) with constraints set at, *a* = [0.1–150], *b* = [1–3] and *c* = [0–1]. In such generalized sigmoidal function, the parameters control a smooth controlled drop-off from a peak value toward an asymptote (here: zero). The parameter *a* can be thought of as a horizontal scaling factor controlling the eccentricity at which the drop-off begins. Parameter *b* controls the sharpness of the drop, and parameter *c* controls the function's tail flatness. To maintain data independence, only left eyes were used to find fit parameters.

### Estimation of D_0_ and Density Profiles for Centrally Obscured Imagery

A separate data set of 57 fully annotated foveal AOSLO images made available from different laboratories (Active Perception Lab, Rochester, NY, USA; Advanced Ocular Imaging Program, Milwaukee, WI, USA; Roorda Lab, Berkeley, CA, USA)^10^^,^^19^ was used to study the predictive value of our fit functions. In these images, a central circular area with varying radii (25, 75, 125, 175 µm) was obscured and data only outside this mask was analyzed (Fig. 7A). To reconstruct density profiles inside the occluded area, first D_0_ was estimated and subsequently function (1) was fit to the density profile outside the occlusion area (Fig. 7B).

D_0_ was estimated by using a linear regression computed between local density z-score and D_0_ in our complete normative data set (Fig. 6A). The local density *z*-score was defined as difference between the individual and group average radial profile expressed in z-scores, as found in a 50 µm wide ring adjacent to the occlusion. Varying the ring width did not improve the regression. D_0_ of occluded eyes was then estimated by plugging their local density *z*-scores into the regression function.

Next, a sigmoid function of the form (1) including the now estimated D_0_ was fitted to the visible density profile outside the occlusion area. As seed, the fit parameters {a, b, c} were set to the group average values of our dataset and then refined for each eye by the same method as described above (Matlab function “fit”, method: Nonlinear least squares; robustness: bisquare; with constraints set at, a = [0.1–150], b = [1–3] and c = [0–1]). This method ensured that each eye received individual parameter sets defining their profile functions. The difference between the estimated profile and actual profile function was used to report estimation errors.

### Statistical Analysis

All statistical analysis was performed in MATAB R2024b. Correlations between fellow eyes and intra-eye metrics were calculated using the *F*-test (Matlab: regress). Similarity between nasal, temporal, superior, inferior, horizontal and vertical meridian density profiles was assessed by a two-sample *t*-test (Matlab: ttest2).

### Data and Code Availability

The MATLAB annotation tool ConeMapper can be downloaded at: https://github.com/ukb-aoslo/ConeMapper. Fully annotated image data can be downloaded at Mendeley. DOI: 10.17632/m5nkpb8phv.1.

## Results

**Figure 2.**
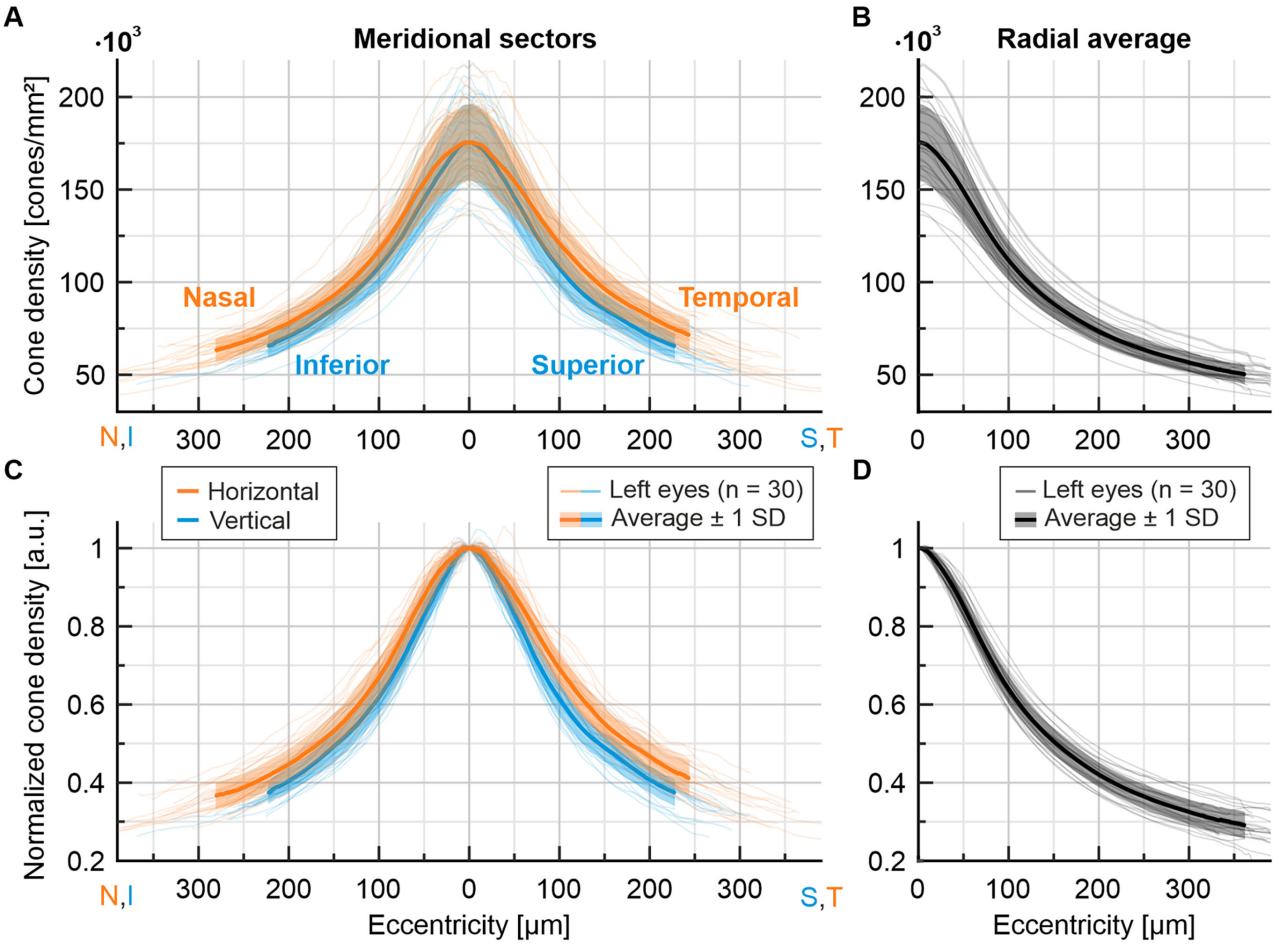
Foveolar cone density profiles. **(A)** Cone density profiles for horizontal (*orange*) and vertical (*blue*) meridional sectors. Inferior and nasal retina is shown on the left, superior and temporal retina on the right. **(B)** Radially averaged cone density profiles. **(C)** Meridional profiles normalized by D_0_. **(D)** Normalized radially averaged profiles. In all panels, individual data are *thin lines*, group average and standard deviation are the *thick lines* and *shaded areas*.

We analyzed a dataset comprising 60 eyes from 30 healthy participants, each with a fully resolved central cone mosaic (Supplementary Fig. S1). All two-dimensional density maps demonstrated a steep increase in cone density toward the foveal center. Fellow eyes presented similar densities and iso-density contour shapes (Supplementary Figs. S2, S4). Because of symmetry between fellow eyes, topographical analyses were performed for left and right eyes separately, and only the results for left eyes are presented here (Fig. 2). D_0_ was on average ± one standard deviation, 175,470 ± 20,540 cones/mm^2^ (range 136,000–216,210 cones/mm^2^), average PCD was 178,700 ± 21,750 cones/mm^2^ (range 137,380–221,060 cones/mm^2^). D_0_ was, on average, 1.8 ± 1.25% lower than PCD. The standard deviation of radially averaged cone density decreased to 5210 cones/mm^2^ at 300 µm eccentricity, two-dimensional density z-score maps are shown in Supplementary Figure S3. A significant difference (two-sample *t*-test, *P* < 0.05) between horizontal and vertical density profiles was observed. There was no statistically significant difference between the superior versus inferior or nasal versus temporal quadrant cone density (two-sample *t*-test). However, a slight trend toward higher cone densities in the temporal quadrant compared to the nasal quadrant was observed. Vertical profiles were on average 18.7% ± 14.7% steeper than horizontal profiles. Cone density dropped to 50% of D_0_ at 143 µm eccentricity along the vertical meridian and at 171 µm eccentricity along the horizontal meridian. Radial profiles reached 75%, 50% and 30% of D_0_ at 72, 151 and 334 µm eccentricity, respectively. Radial profiles therefore more closely resemble vertical profiles than horizontal profiles, suggesting that the overall two-dimensional retinal topography generally resembles the vertical meridian. Radial density profiles displayed a steep drop with a maximum slope of −870 ± 184 (cones/mm^2^)/µm (range −490 to −1230 (cones/mm^2^)/µm) at an average eccentricity of 51 ± 8 µm (range 32–70 µm). We find a significant positive correlation between axial length and D_0_ expressed in angular units, cones/deg^2^ (*R*^2^ = 0.18, *P* = 0.02). A nonsignificant negative trend was present when D_0_ was expressed in linear units, cones/mm^2^ (*R*^2^ = 0.08, *P* = 0.123). Angular density and inter cone distance profiles are presented in Supplementary Figure S5. Data for both eyes, including density values in angular units as well as inter cone distances, are available in Supplementary Table S1.

**Figure 3.**
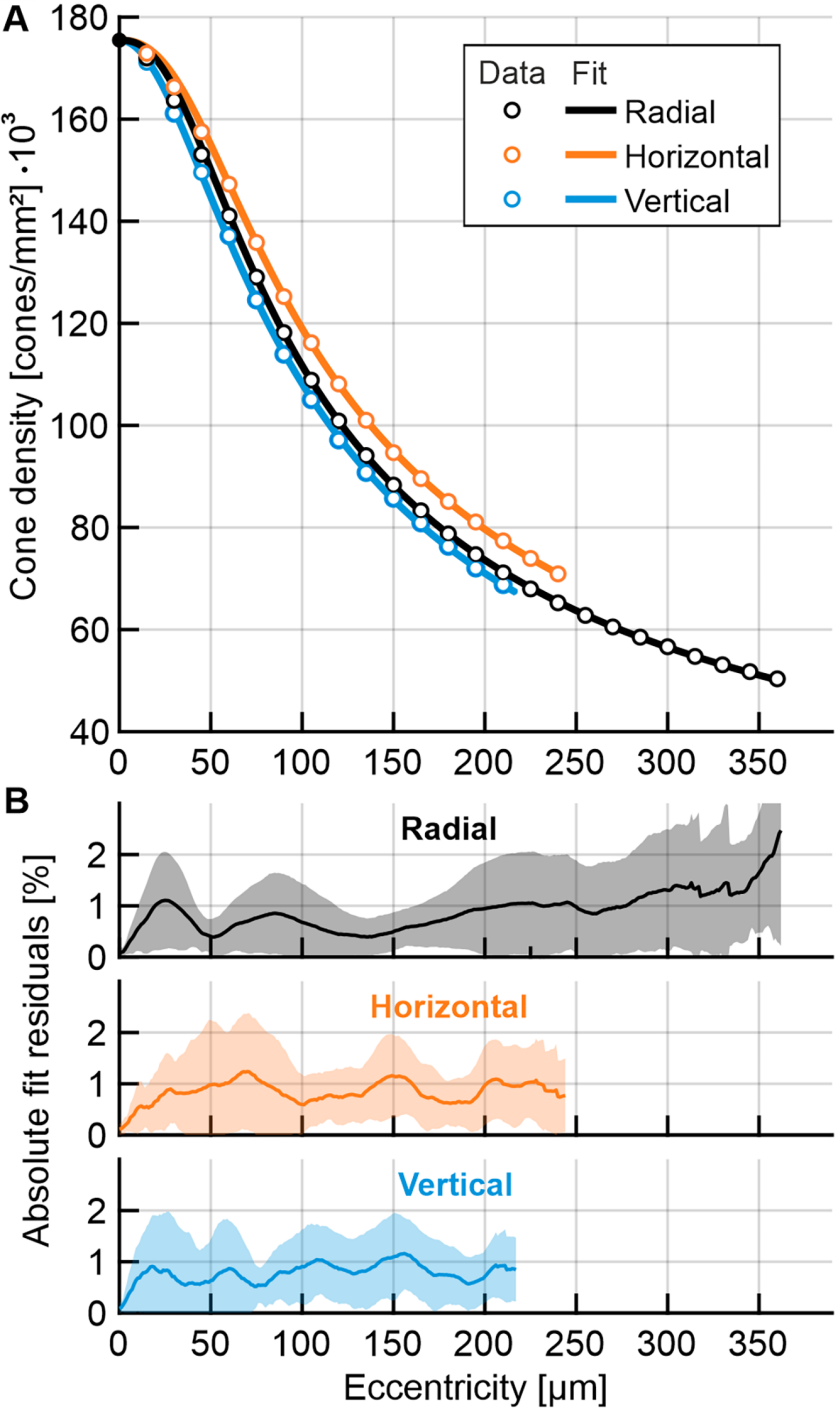
Density profile modeling. **(A)** Radial (*black*), horizontal (*orange*), and vertical (*blue*) average density profiles of 30 left eyes were fitted by a four-parameter sigmoid decay function (see Methods and Table 2). **(B)** Errors are calculated as the difference between individual cone densities and their individually fitted profile function. The *line* represents the group's average error, *shaded regions* denote ±1 standard deviation.

To mathematically describe the cone photoreceptor topography of the normal human foveola, radial, horizontal and vertical profiles averaged across all eyes were fit by four-parameter sigmoid functions (Fig. 3A). For all meridional profiles, density typically drops increasingly to an inflection point where the slope is maximal, from which it decelerated until a modest slope, finally presenting a nearly asymptotically convergence to zero, resembling a sigmoid curve. Best fit parameters to the group average density data are presented in Table 2. Errors are calculated as the difference between individual cone densities and their individually fitted profile function. The average fitting error within the analyzed area was found to be within two percent (Fig. 3B).

**Table 2.** Fit Parameters for Horizontal, Vertical, and Radial Average Density Profiles

Profile	D_0_	a	b	c
Horizontal	175,500	61.95	2.469	0.2680
Vertical		59.11	2.012	0.3568
Radial		55.50	2.453	0.2726

**Figure 4.**
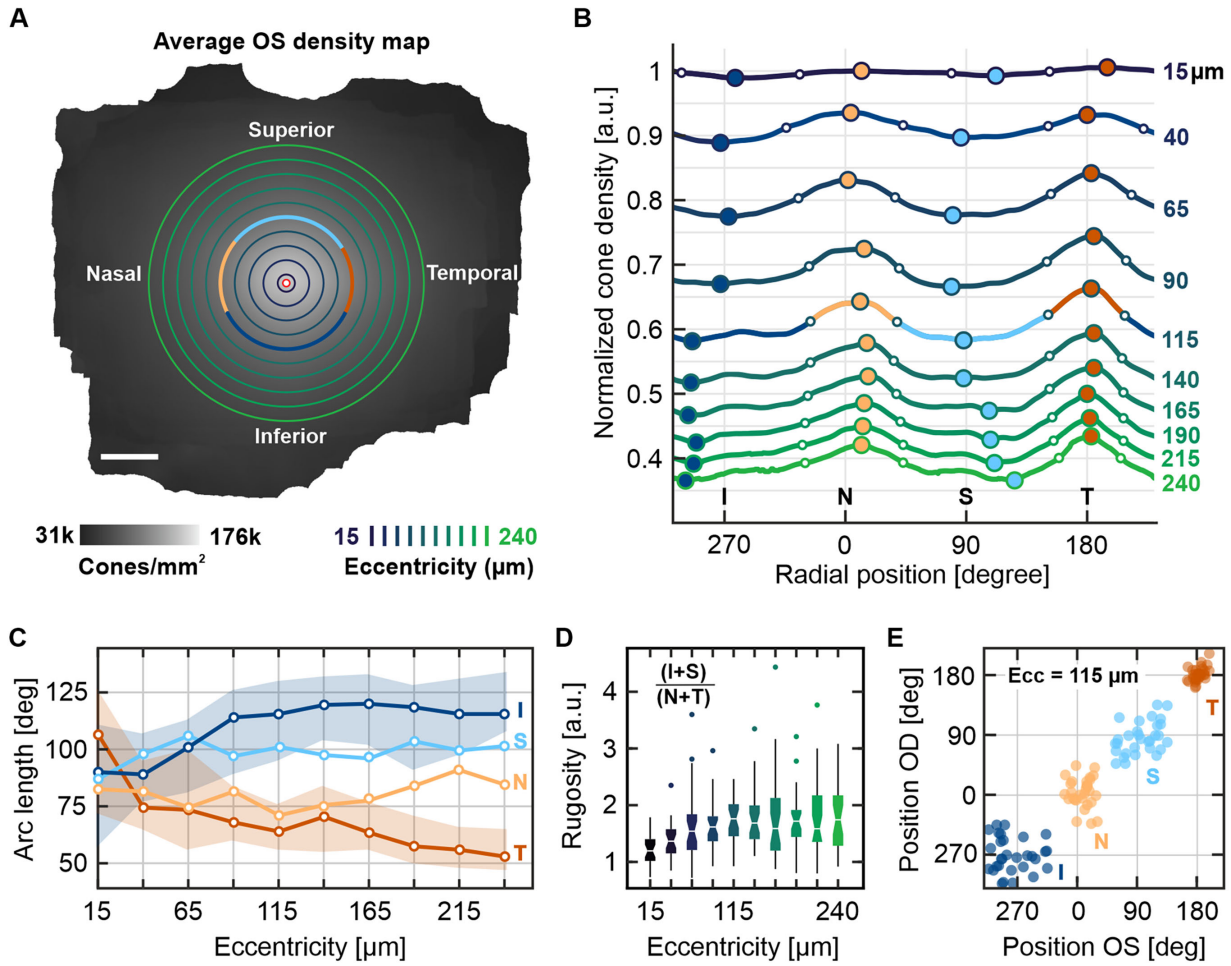
Circular density distribution and rugosity analysis. **(A)** Radial cone density profiles were extracted from the cone density map at various eccentricities (15, 40, 65, 90, 115, 140, 165, 190, 215, and 240 µm, indicated by the *dark blue* to *light green circles*). Shown here in *grayscale* is the average cone density map of 30 left eyes in fundus orientation. The CDC is marked by a *red-white circle*. **(B**) Radial profiles were normalized by D_0_ and used to find peaks (*yellow* and *red*) and troughs (*light* and *dark blue*). *White circles* mark the half-height between neighboring peaks and troughs and define their respective widths. One such profile, at 115 µm eccentricity, is indicated in **A** and **B** as an example. **(C)** Peak and trough widths analyzed across all eyes. *Shaded areas* are interquartile ranges (omitted for better visibility for similar superior and nasal data). **(D)** Rugosity, defined as the ratio of trough to peak widths, per eccentricity as boxplots. **(E**) Radial peak and trough positions of left (OS) and right (OD) eyes at 115 µm eccentricity.

Because of the anisotropy between vertical and horizontal density profiles, we examined the rugosity of the two-dimensional topography, defined as its deviation from rotational symmetry. For this, we took all individual and an averaged cone density map and extracted the D_0_-normalized cone density along ten circular radii (15–240 µm eccentricity). Such circular profiles were analyzed along their angular position (0° = nasal, 90° = superior retina). We identified peaks and troughs in these profiles, and assessed the angular widths between their respective half-height points (Fig. 4). The average circular profile at 115 µm eccentricity had peaks at 11° and 183° (close to the horizontal meridian), and troughs at 99° and 257° (close to the vertical meridian). With increasing eccentricity, nasal and temporal peak widths decreased while the trough width increased correspondingly (Fig. 4C). The median peak widths were 80° and 64° for nasal and temporal retina, whereas superior and inferior troughs were 100° and 116° wide between 65 and 240 µm eccentricity. The topographical rugosity was defined as the combined trough/peak width ratio. A rugosity factor greater than one indicates the presence of a horizontal ridge of elevated cone density. We found an average rugosity range between 1.35 and 1.65 for the eccentricities studied (Fig. 4D). There was no significant correlation between left and right eyes rugosity. Similarly, no correlation between fellow eyes peak and trough positions was observed. The temporal peak position was the least scattered around its average position (Fig. 4E).

**Figure 5.**
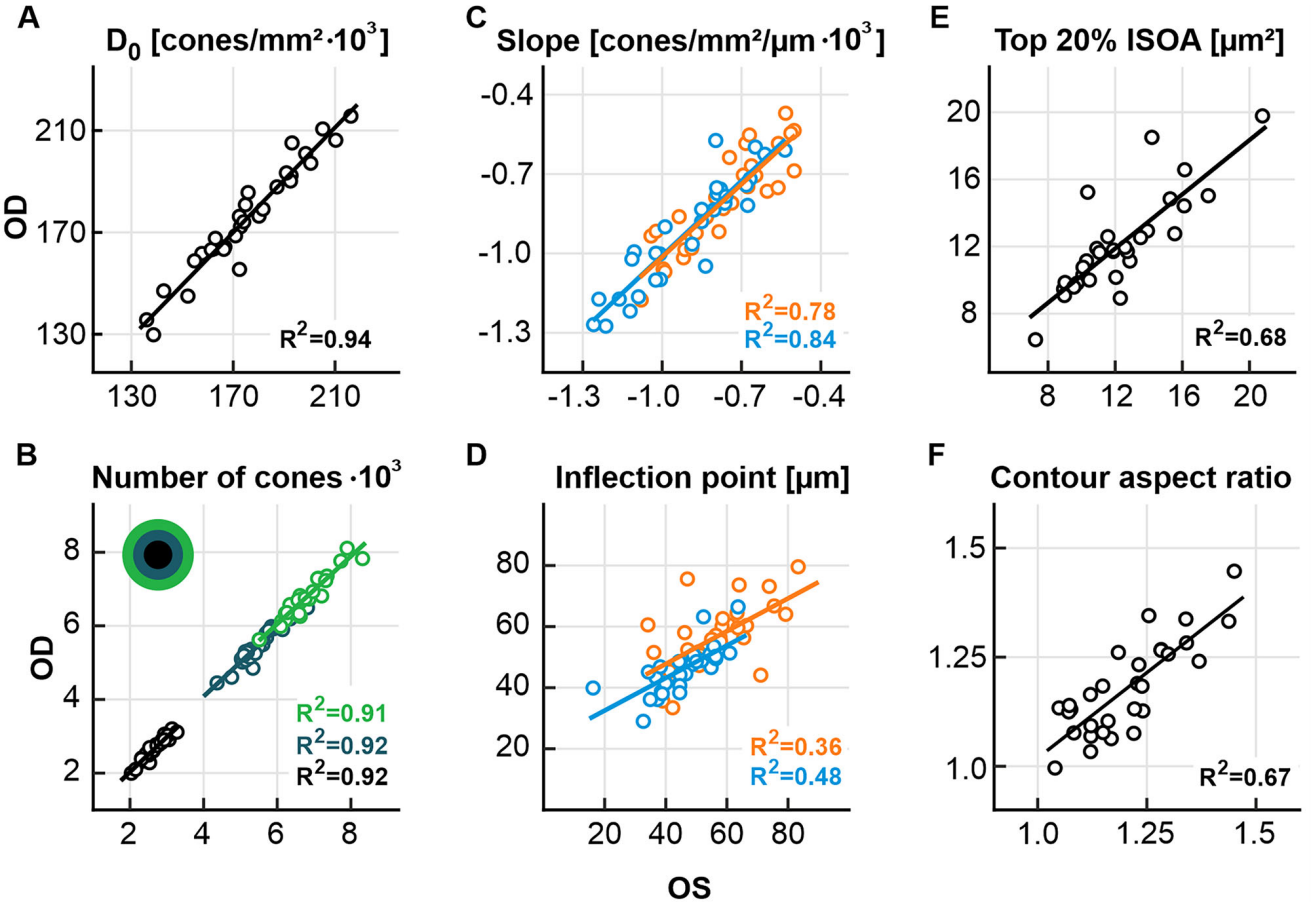
Fellow-eye correlations. **(A)** Fellow-eye correlation of D_0_. **(B**) Number of cones in a circle (radius = 75 µm, black) and two ring shapes (ring1: inner radius 75 µm, outer radius 150 µm, *dark green*; and ring2: inner radius 150 µm, outer radius 225 µm, *light green*) around the CDC. **(C, D)** Maximum slope and inflection point of the horizontal (*orange*) and vertical (*blue*) profiles. **(E)** Iso-density contour area (ISOA) covered by the top 20% cone densities values. **(F)** Aspect ratio of the horizontal/vertical diameter for a contour of the mean density at 115 µm eccentricity. In all panels, left eyes (OS) are on the abscissa. All correlations display statistical significance (*F*-test, *P* < 0.001).

We generally observed high intra-individual symmetries of foveolar cone topography metrics. D_0_ was highly correlated between fellow eyes (*R*^2^ = 0.94, *P* < 0.001, Fig. 5A). Likewise, the total number of cones within a circular area (radius 75 µm) and two distinct ring shapes (ring1: inner radius 75 µm, outer radius 150 µm and ring2: inner radius 150 µm, outer radius 225 µm), centered on the CDC were all similarly correlated (*R*^2^ > 0.91, *P* < 0.001, Fig. 5B). The maximum slope of the horizontal (*R*^2^ = 0.78, *P* < 0.001) and vertical (*R*^2^ = 0.84, *P* < 0.001) profiles were highly correlated between fellow eyes, while the inflection point (eccentricity with maximum slope) displayed a weak correlation (horizontal profile: *R*^2^ = 0.36, *P* < 0.001, vertical profile: *R*^2^ = 0.48, *P* < 0.001, Figs. 5C, 5D). The area of the iso-density contour where cone density dropped to 80% of D_0_ was highly correlated (*R*^2^ = 0.68, *P* < 0.001, Fig. 5E) between fellow eyes as well. As a complementary analysis to the rugosity investigation, the average circular density at 15, 40, 65, 90, 115, 140, 165, 190, 215, and 240 µm eccentricity was computed and used to produce iso-density contours. An analysis of the contour shapes, when expressed as their aspect ratio (horizontal divided by vertical axis length) displayed the strongest correlation for the contour of the average density at 115 µm eccentricity (∼59% of D_0_, *R*^2^ = 0.67, *P* < 0.001, Fig. 5F).

**Figure 6.**
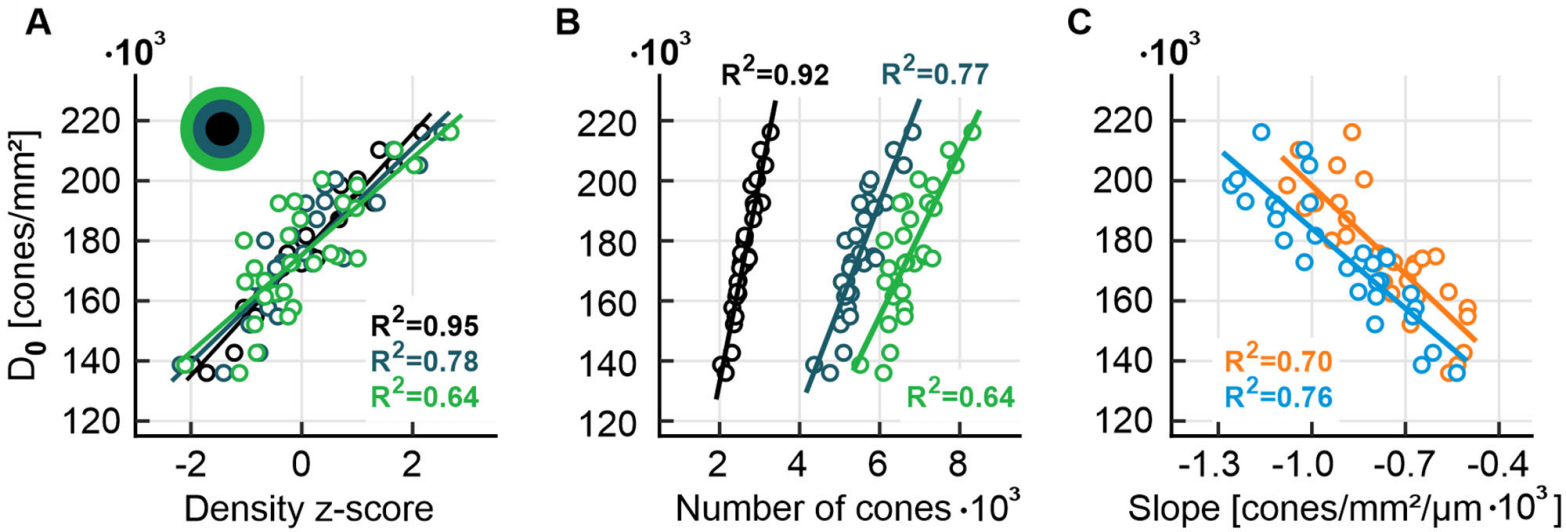
Correlation of central cone density, D_0_, with topographical metrics. **(A, B)** Correlations of the density z-score and number of cones in a circular and two ring areas around the CDC (analysis areas as described in Fig. 5) with D_0_. **(C)** Horizontal (*orange*) and vertical (*blue*) profile slope correlation with D_0_. All correlations display statistical significance (*F*-test, *P* < 0.001).

We observed a significant correlation between D_0_ and other mosaic quantities. For example, a very high correlation was observed with the density *z*-score within a concentric circular area of 75 µm radius (*R*^2^ = 0.95, *P* < 0.001), as well as in progressively larger circular rings surrounding it (*R*^2^ = 0.78, *P* < 0.001, ring1 and *R*^2^ = 0.64, *P* < 0.001, ring2) (Fig. 6A). Similarly, the number of cones within circle1 and ring1 presented very high correlations (*R*^2^ = 0.92, *P* < 0.001 and *R*^2^ = 0.77, *P* < 0.001, respectively) and within ring2 a high correlation (*R*^2^ = 0.64, *P* < 0.001) (Fig. 6B). Furthermore, the maximum slope of the density profile was well correlated to D_0_, with *R*^2^ = 0.7, *P* < 0.001 and *R*^2^ = 0.76, *P* < 0.001, for horizontal and vertical profiles, respectively (Fig. 6C). These observations motivated an attempt to estimate D_0_ and reconstruct portions of the full density profile in incomplete foveal imagery.

**Figure 7.**
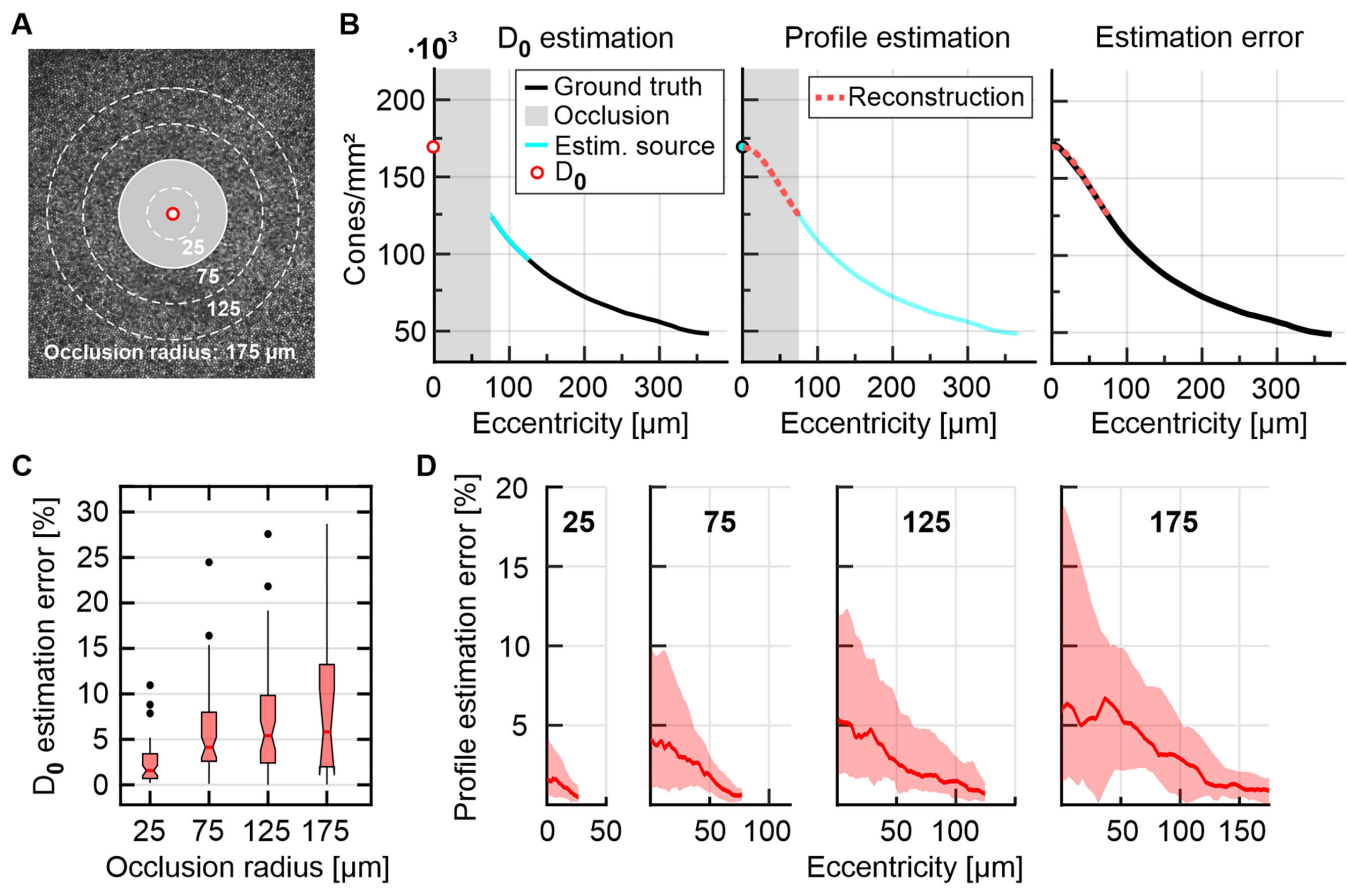
Estimation of cone density in incomplete imagery. **(A**) In 57 eyes, central circular image areas and their data were occluded (*gray disk*, example radius 75 µm). **(B**) To recover radial profiles, first D_0_ was estimated based on the individual eye's average density *z*-score in a 50 µm ring adjacent to the occlusion radius (*cyan line*). Then the full profile was estimated via fitting our profile model to the visible data and the estimated D_0_. **(C**) Absolute estimation error of D_0_ for different occlusion radii. **(D**) Median (*thick lines*) and 16th to 84th percentile (*colored area*) profile estimation error within the occlusion zone.

To investigate the potential for estimating D_0_ and foveal cone density profiles in eyes with unresolved central photoreceptor mosaics, a dataset of 57 fully annotated foveal images acquired in different AOSLO instruments and laboratories was analyzed. Central circular areas of different radii (25, 75, 125, and 175 µm) were occluded to simulate unknown (e.g., unresolved) photoreceptor areas (Fig. 7A). D_0_ was estimated by linear regression of the average cone density z-score in a 50 µm ring sector adjacent to the occluded area (Figs. 7B, C). A median absolute estimation error of 1.6%, 4.2%, 6%, and 6.8% for the 25, 75, 125, and 175 µm radii occlusion zones was observed across all eyes. Including all topography metrics (density *z*-score, number of cones, density slope) in a multiple linear regression did not improve the estimation. After D_0_ estimation, our density profile model was fitted to the visible data (Figs. 7B, C). On average, all profile estimation errors had their maximum value at D_0_ and decreased monotonically to the occlusion border (Fig. 7D). For larger occlusion radii, the central 50 µm accounted for an estimation error of about ∼5%. Individual eyes that exhibited significant deviations from the typical sigmoidal shape, particularly near the CDC, demonstrated reduced estimation accuracy.

## Discussion

In this study, we quantified the in vivo photoreceptor mosaic of the human foveola in 60 eyes of 30 healthy participants from ∼2° AOSLO images in which all cone locations were annotated. We found that cone density of the normal foveola can be modeled by a sigmoidal decay function. We presented an approach to estimate central cone density in cases of incomplete image data (e.g., when the area isn't fully resolved).

**Figure 8.**
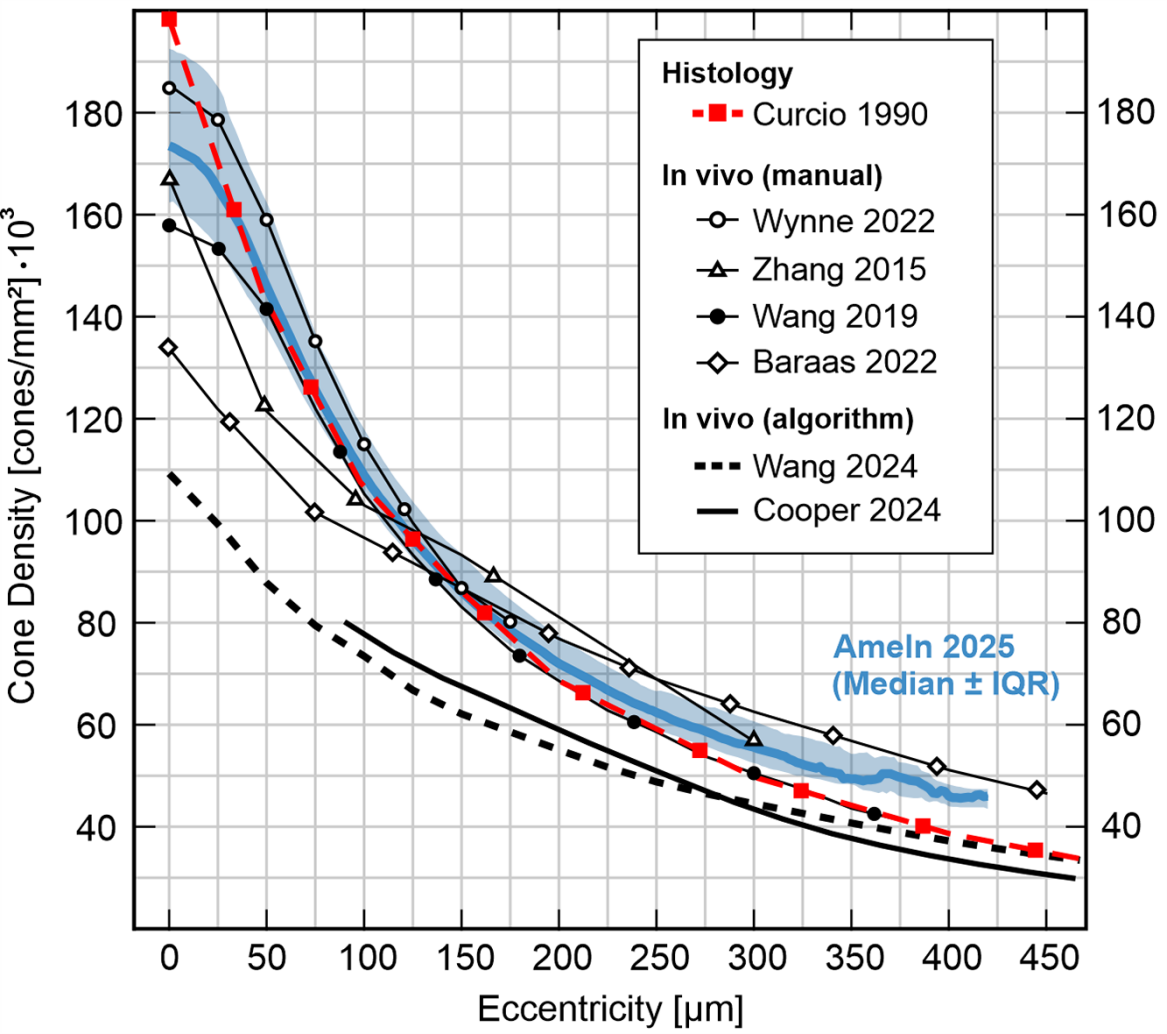
Ex vivo and in vivo foveolar cone topography compared. The current standard, histology, is the *red dashed line* with square markers (*n* = 7). In vivo data, using cone center annotation including manual corrections (*lines* with different markers, see legend), or other algorithmic approaches (*solid* or *dashed lines* without markers), are based on different numbers of eyes (compare Table 1). Our data is represented by the *thick blue line* with the shaded area indicating the interquartile range (*n* = 30).

The cone density profiles observed in our study present a sigmoid-like form and are generally comparable to those found in previous studies (Fig. 8).^10^^,^^23^^,^^34^^,^^35^ The strength of our study lies in the extraction of density profiles with much finer resolution compared to the spaced ROIs in other studies^12^^,^^18^^,^^36^ while capturing the full extent of the density drop of the foveola. Notably, studies using fully automated cone density estimation methods, such as Yellot's ring analysis, or a peak finder algorithm report considerably lower cone densities within the foveola.^37^^,^^38^

Our four-parameter model can be fitted to the density profiles of individual eyes with an average error of less than 2%, yielding smoothed and improved profiles along the major meridians as well as the full radial profile. This allowed us to estimate cone density in artificially occluded imagery via *z*-score regression, producing average median estimation errors between 1.6% to 6.8% for D_0_ and smaller errors for full profiles. We found a strong correlation between cone density outside the foveola and D_0_; however, individual eyes may deviate—especially at the topographical center of the retina. We found evidence for more cone density variability in central areas (see Figs. 2A, 2B). Given that we average over more cones at larger eccentricities in our analysis, it is thus likely that the D_0_ estimation error is predominantly driven by higher variability of the central mosaic. These observations are in line with the assumption that multiple factors influence cone migration. We see evidence of at least two distinct processes; one may initiate the overall cone migration into the fovea, possibly establishing a baseline cone density level. Another process subsequently fine tunes the cone density within the foveola. This observation is in line with three phases of cone migration that have been previously described in histological samples, of which two involve the foveal center, initially in prenatal and later in postnatal time.^1^^,^^2^ It furthermore accommodates an increased topographical variability towards the center.^12^^,^^18^ That we find a correlation between axial length and central cone density is consistent with reports by Wang et al.,^10^ supporting a mixed model of eye growth that involves both equatorial stretching and global expansion of the eyeball.

A comparison of the average meridional and radial fits shows that the overall radial profile closely aligns with the vertical profile. In contrast, the horizontal profile is distinctly elevated, suggesting a horizontal streak with increased cone density. A similar observation was made at larger eccentricities, meaning that this particular shape already starts in the very center and is conserved even at higher eccentricities.^12^^,^^39^ This is in line with horizontal visual streaks being common in a variety of primate species, a remnant of a retinal morphology thought to aid predator detection along the horizon.^40^^–^^42^

Previous studies examining the cone mosaic across larger sectors have typically been limited to comparisons between the horizontal and vertical meridians, providing only sparse information about the full radial profile. This limitation stems from extended imaging durations, existing constraints in automated analysis, and the exponentially increasing human effort required for a more comprehensive assessment. Although efforts are underway to map cone density across larger two-dimensional areas of the eye, existing data remains limited and largely restricted to spaced ROI analysis.^18^^,^^37^ Based on our findings, we suggest that cone densities measured at locations offset from the major meridians should not be directly compared to meridional densities at the same eccentricity. Our two-dimensional rugosity analysis (see Fig. 4) can be used as guideline for relative off-meridional densities.

As part of an ongoing effort to establish reliable and general standards for topographic data analysis, we used D_0_, the cone density at the CDC, to report central cone density.^19^^,^^23^ D_0_ ranged from 136,001 to 216,209 cones/mm^2^, in alignment with previously reported central foveal densities and ranges (see Table 1). Cone density at the CDC was on average 1.7% lower than PCDs (range 0.2%–6%), consistent with earlier reports.^19^^,^^43^ This differences may be taken into consideration when comparing density results from different studies.^43^ It is noteworthy that histology based studies reported much higher variability in peak cone density, especially due to a singular case of ultra-high density exceeding 300 thousand cones/mm^2^ (Fig. 8).^12^ Most likely this is due to tissue shrinkage, resulting in tighter cone packing,^12^ and not because of limited imaging resolution in in vivo approaches.^18^

Furthermore, different cone annotation strategies affect the reported density variations. Our annotation approach considers photoreceptor mosaic characteristics and imaging techniques. Although the cone mosaic in the foveola is mostly hexagonally packed, the specific imaging modality can alter its appearance. In confocal AOSLO images, cones vary in brightness and may appear dark despite normal function.^44^ Multiple smaller foveal cones may blur together to appear as a larger or slanted singular cone, and rods near the fovea (0.5°–0.75°) integrate into the mosaic, disrupting its regularity.^12^^,^^45^^,^^46^ To address this, we manually refined automatic annotations, assuming a near hexagonal arrangement to mark dark or merged cones—similar to Wang et al.^10^ Early rods detected as cones were excluded based on size considerations within the mosaic of surrounding cones. Adopting a consistent annotation strategy may reduce interstudy and intergrader differences, as observed by Wynne et al.^19^ Importantly, this method needs to be applied with care in cases of retinal pathologies where structural changes may influence the overall photoreceptor appearance.

Similarly, we advocate for a density mapping method based on the pixel-wise Voronoi area of the 150 nearest cones.^23^ This approach offers a size-adaptive analysis area that accommodates the substantial variations in local cone density. By incorporating the nearest cones rather than a square window, the method ensures that only the most relevant cones contribute to each analyzed location. The choice of 150 cones, although arbitrary, is informed by striking a balance between robustness against small annotation jitter and preserving local density variations. A recent study that systematically changed analysis window size and shape confirmed this to be a well-balanced approach.^47^ Applying these annotation and density computation techniques could enhance the comparability of studies reporting photoreceptor density values. Approaches to directly estimate cone density by Yellott's ring analysis would allow circumventing of the difficulties of proper cone annotation, but they do currently present a lack of confidence within the foveal center.^38^^,^^48^

To assess the similarity of the photoreceptor mosaic in fellow eyes, we analyzed inter-eye relationships of multiple topography metrics (Fig. 5). We observed strong inter ocular symmetry, including a very strong correlation of D_0_, as well as good correlations of iso-density contour area sizes and contour roundedness (Figs. 5A, 5E, 5F). Our findings are consistent with previous reports of high inter-eye symmetries of these metrics.^20^^,^^23^ Extending these previous findings we found high correlations of the number of cones within distinct donut shaped areas around the CDC as well as the meridional maximum density profile slopes (Figs. 5B, 5C). The inflection point of the maximum density slope presents low (horizontal meridian) to mild (vertical meridian) correlations (Fig. 5D). Collectively, our analysis confirms that the photoreceptor topography of the foveola is highly similar and to a degree mirror symmetric between fellow eyes.

Investigating the relationship between eye growth and D_0_, we find a positive correlation between axial length and D_0_ when expressed in angular units, and a negative trend in linear units. Our findings are consistent with those reported by Wang et al.,^10^ supporting a mixed model of eye growth that involves both equatorial stretching and global expansion of the eyeball.

Our results will be relevant for studies investigating the treatment of photoreceptors in retinal disease. Today, clinical studies aiming to prevent retinal degeneration in diseases like retinitis pigmentosa, Stargardt's disease, choroideremia, or age-related macular degeneration are underway.^49^^,^^50^ Appropriate clinical endpoints and relevant, sensitive readout parameters are thus much sought after. Visual function like navigation capabilities^51^^,^^52^ and best-corrected visual acuity^24^^,^^25^ are likely not sensitive enough to pick up small but significant changes in consequence of a treatment. Therefore sensitive readout parameters that allow short-time assessment of treatment outcome are desired, and the fine structure and cellular topography of the foveolar center might be a promising candidate.^7^ Our findings can serve as normative reference or benchmark against which diseased retinas can be pitted. In any given eye, expressing cone density as difference from “normal” (e.g., in *z*-scores) would allow quantification and thus qualification of the severity of photoreceptor loss. Considering potential gene restoration treatment^53^^,^^54^, our findings define a target/ceiling a photoreceptor rescue could potentially reach. For cell therapy^55^^,^^56^ and retinal chip treatments,^57^^,^^58^ our data can guide decisions about the ideal photoreceptor topography a transplant should exhibit. Our model can also be applied to estimate the density profile in individual retinas, similarly as is shown in the reconstruction of occluded areas. If the foveal photoreceptor mosaic is damaged only partially with a spared center (e.g., in cases of MacTel),^59^ our approach allows estimation (and thus comparison) of the local cone density this retina would likely have had at the region of interest, were it healthy.

One limitation of our study is the low median age and age range of our participants. Although we do not observe correlations of D_0_ or the number of cones in a central one degree circle with age, it has been previously shown that cone density close to the foveal center declines with age.^60^ Whether this is the case for the foveal center is unknown. Thus larger cohorts drawn from all ages need to be studied in the future to comprehensively describe photoreceptor mosaic changes in the aging retina. Additionally, while cone density serves as a structural marker of retinal health, retinal function can persist in areas with apparent cone loss. Studies using adaptive optics microperimetry have demonstrated that regions with reduced cone density can still exhibit notable visual sensitivity.^26^^,^^59^^,^^61^^,^^62^ Future work should explore the structure-function relationship, combining high-resolution imaging with functional testing to better understand how the local photoreceptor arrangement translates to visual perception of small stimuli in the foveola.

Our study provides a high-resolution quantitative model of foveal cone density, describing the central photoreceptor mosaic with a sigmoid function that captures both meridional density variations as well as a generalized radial profile from in vivo data. This model enhances our understanding of normal cone distribution and offers, together with a linear regression-based estimation of D_0_, a method for reconstructing missing central density values. By prioritizing comparability across studies and establishing a normative reference database, our findings lay the groundwork for future research and clinical applications in high-resolution retinal imaging of the human foveola.

## Supplementary Material

Supplement 1
