# Supplementary material for: In Vivo Cone Photoreceptor Topography of the Human Foveola
Source: Invest Ophthalmol Vis Sci. 2025 Aug 6;66(11):13. doi: 10.1167/iovs.66.11.13 (PMC12347159; doi:10.1167/iovs.66.11.13)
Supplement: Supplement 1 [file iovs-66-11-13_s001.pdf]

Supplemental information

In-vivo cone photoreceptor topography of the human foveola

Julius Ameln, Jenny L. Witten, Aleksandr Gutnikov, Veronika Lukyanova, Frank G. Holz, Wolf M. Harmening

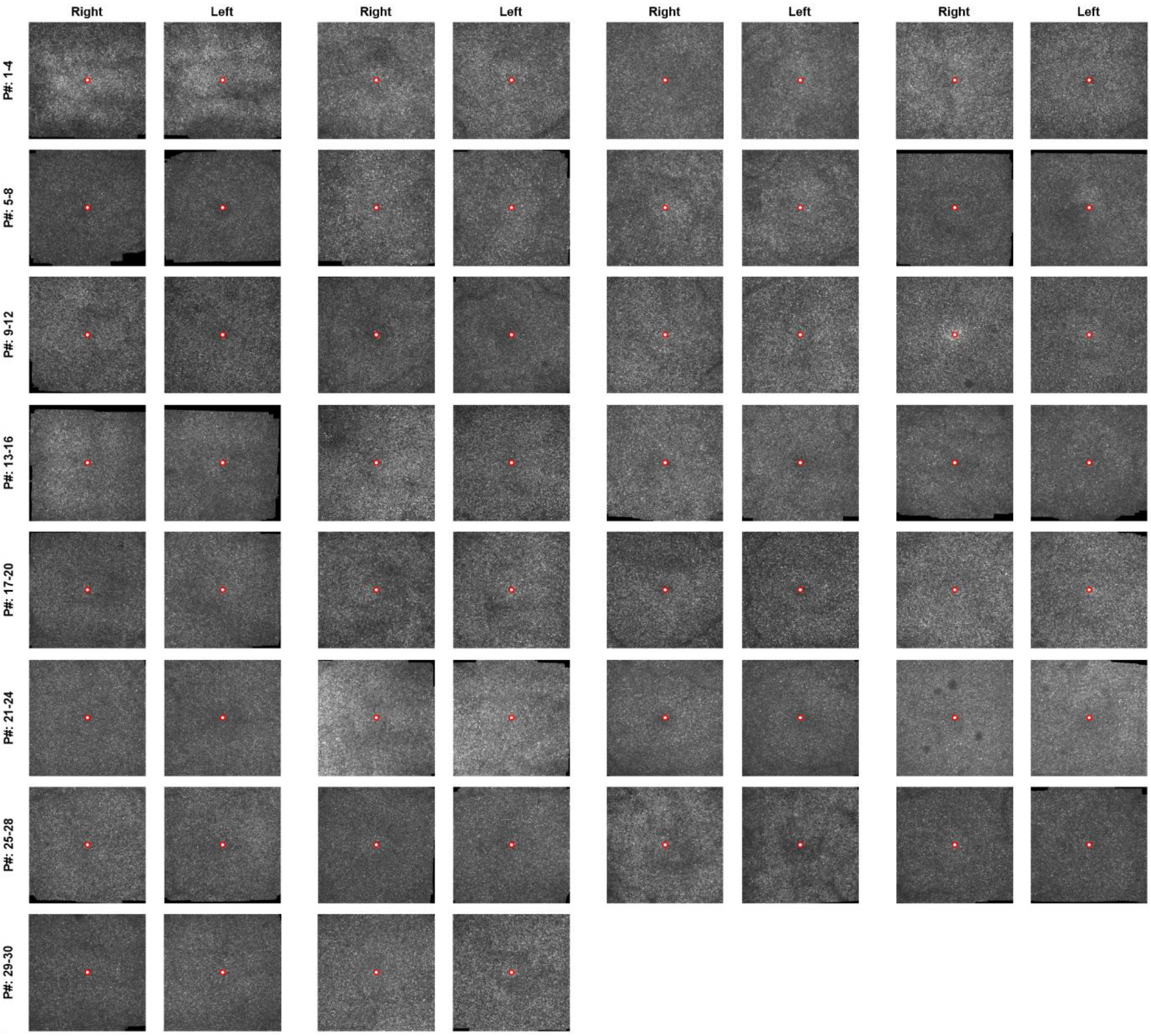

Figure S1 | Confocal AOSLO image crops. Central 500 x 500 µm crops of the larger montages of all 60 eyes of 30 participants (P1-30). All images are in fundus orientation, i.e. superior is at the top and nasal is between two image pairs. Images are centered on the cone density centroid (CDC, red-white circle). Participants are named in an ascending order of D_0, the cone density at the CDC in the left eyes (from lowest to highest, top left to bottom right).

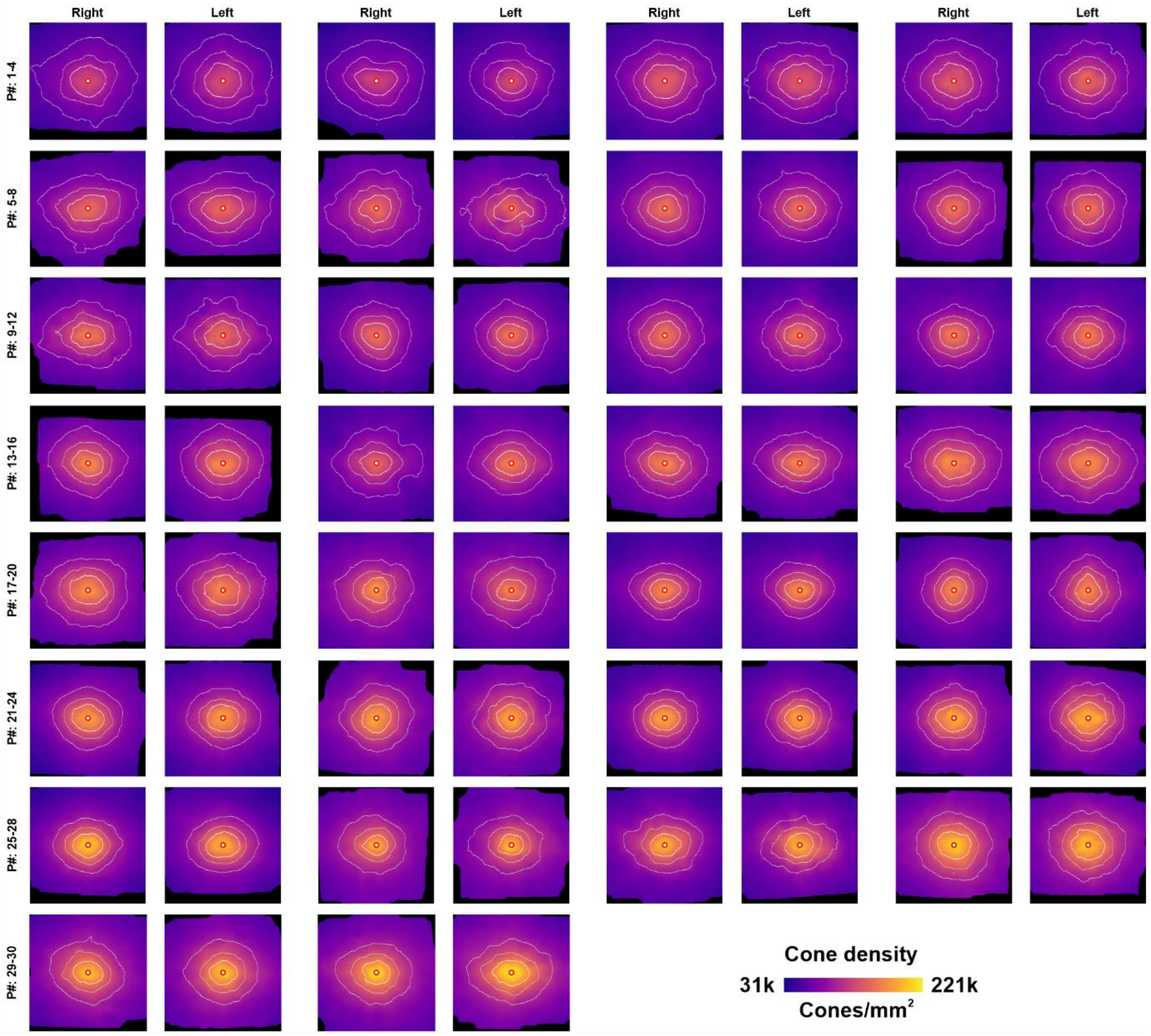

Figure S2 | Cone density maps. Color is absolute cone density. White lines indicate iso-density contours at 80%, 65% and 50% of D_0. Image size and arrangement identical to Figure S1.

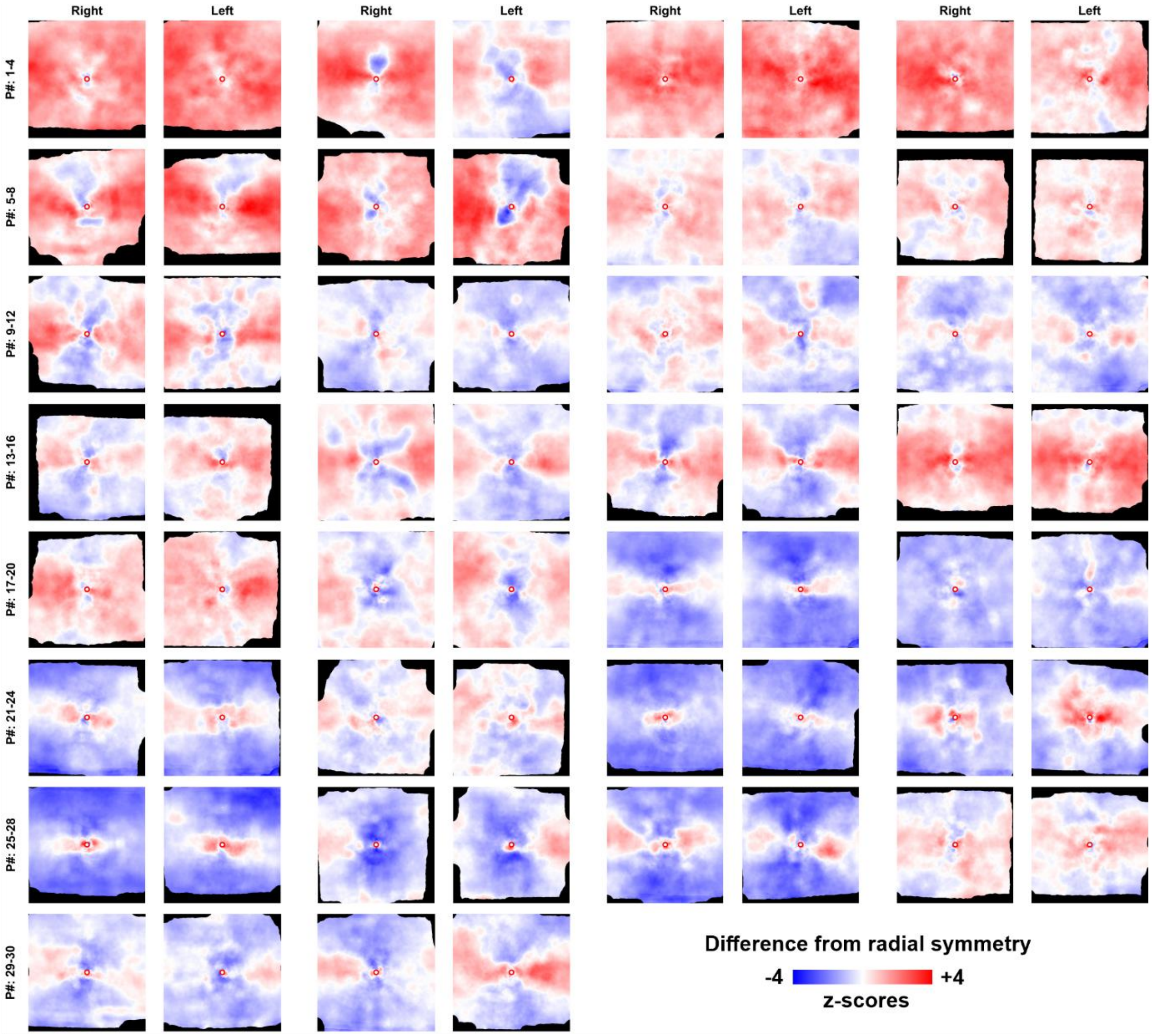

Figure S3 | Deviation from radial symmetry. Plotted is the difference (in z-scores) from a theoretical radially symmetrical profile which was constructed from the average radial density profile. Individual density maps were first normalized by D_0. Image size and arrangement identical to Figure S1.

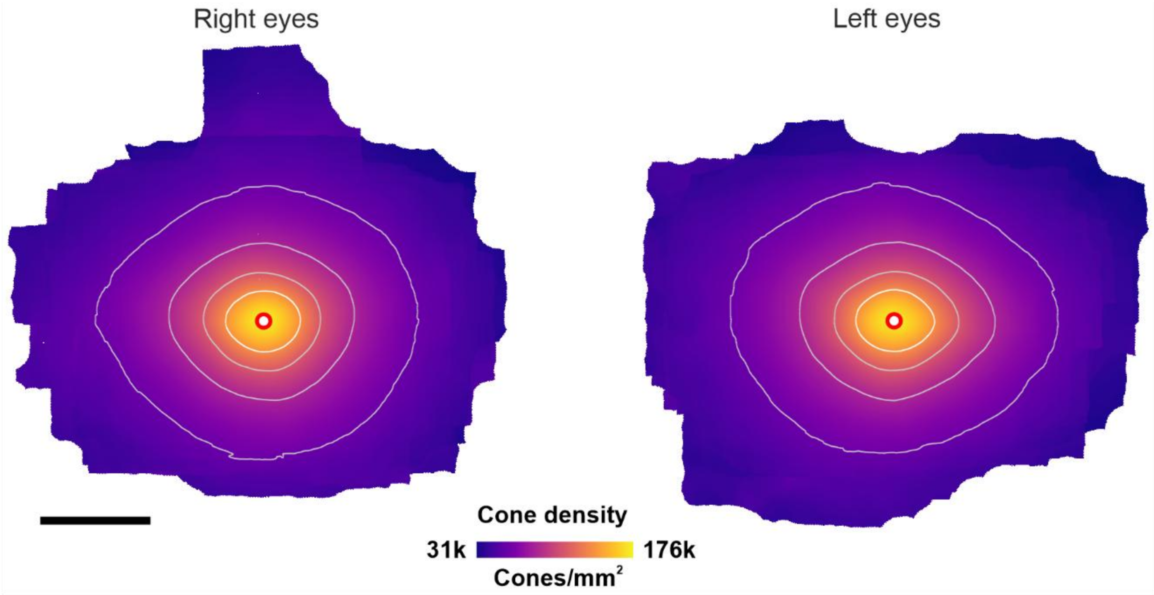

Figure S4 | Average cone density maps of all right and left eyes. Iso-density contour lines are at 80% (bold white), 65%, 50% and 35% of D_0. Scale bar is 200 µm.

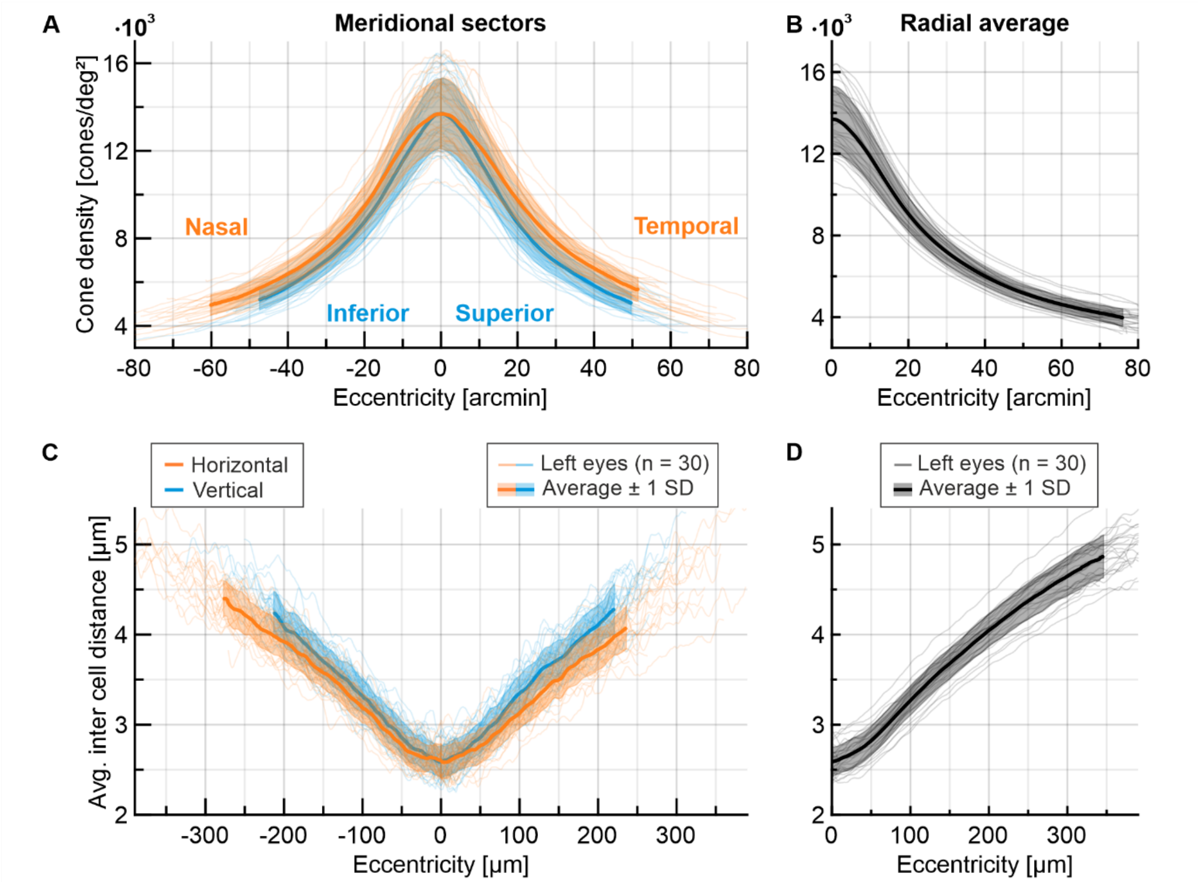

Figure S5 | Angular cone density and inter cone distance. A: Cone density profiles in angular units for horizontal (orange) and vertical (blue) meridional sectors. Inferior and nasal retina is shown on the left, superior and temporal retina on the right. **B**: Radially averaged cone density profiles. **C**: Meridional profiles of average inter cone distance. **D**: Radially averaged inter cone distance profiles. In all panels, individual data are thin lines, group average and standard deviation are the thick lines and shaded area.

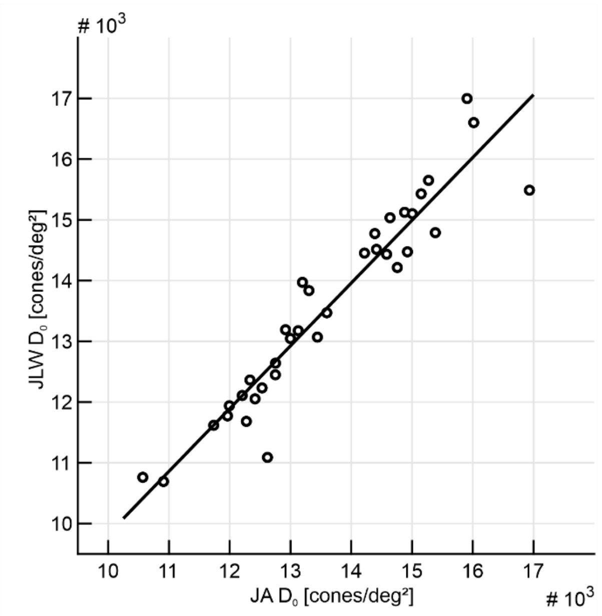

Figure S6 | Assessment of grading reproducibility. Manual annotation correction was performed by authors JA and JLW in a subset of the data (35 eyes from 18 participants) and compared. Cone density at the D_0 is expressed in angular units. The average absolute difference between graders was 2.8 ± 2.7 %.

Table S1. Participant statistics and cone mosaic metrics. All metrics are measured or calculated as described in the studies method section.

Participant#	Participant ID	Sex	Age	Eye#	Eye	Axial length	RMF	PCD [cones/ mm²]	D_0 [cones/ mm²]	PCD [cones/ deg²]	D_0 [cones/ deg²]	Peak-ICD [µm]	D_0-ICD [µm]	Max. slope [cones/mm²] /µm	Inflection point [µm]	#cones in 1 degree dia.	Top 20% cone density contour area [µm²]	Contour roundedness [h/v]	2D rugosity
1	BAK1101	f	33	1	OS	24.43	296.1	137382	136001	12042	11921	2.48	2.84	-565	61	6808	17544	1.07	0.98
				2	OD	24.43	295.2	136110	135681	11862	11824	2.54	3.11	-575	53	6561	15029	1.12	0.7
2	BAK1088	f	22	3	OS	23.95	290.1	140345	138705	11811	11673	2.44	2.82	-614	42	6106	10362	1.25	1.87
				4	OD	23.58	284.7	131147	129792	10630	10520	2.51	3.03	-492	55	5958	15237	1.35	1.34
3	BAK8015	m	32	5	OS	24.87	301.8	145640	142715	13265	12999	2.19	2.94	-608	70	7525	20773	1.23	1.6
				6	OD	25	304.1	149123	147038	13790	13598	2.29	2.84	-643	69	7792	19783	1.19	2.37
4	BAK1035	f	25	7	OS	24.81	297.2	157822	152194	13940	13443	2.19	2.79	-722	58	7313	14187	1.17	2.01
				8	OD	24.9	298.5	148717	144985	13251	12919	2.35	2.82	-593	67	7401	18503	1.06	1.54
5	BAK1068	f	18	9	OS	22.03	261.3	157017	154803	10721	10570	2.28	2.94	-677	56	6196	16109	1.45	1.93
				10	OD	22.17	262.1	159853	158817	10981	10910	2.12	2.85	-718	52	6269	14425	1.45	1.6
6	BAK1014	f	28	11	OS	23.26	278.2	160464	157707	12419	12206	2.11	2.65	-701	38	6678	11885	1.24	1.2
				12	OD	23.5	280.8	162792	161715	12836	12751	2.21	2.67	-702	43	6956	11706	1.13	1.3
7	BAK1089	f	18	13	OS	23.74	284.6	162291	161398	13145	13073	2.33	2.68	-732	50	6987	11564	1.22	1.27
				14	OD	23.68	283.9	165013	163209	13300	13154	2.26	2.64	-713	52	7161	12589	1.08	1.05
8	BAK1028	f	31	15	OS	22.68	268.8	165472	162431	11956	11736	2.13	2.6	-774	57	6569	13511	1.05	1.58
				16	OD	22.85	270.8	166801	163552	12232	11994	2.22	2.56	-739	51	6608	12531	1.13	1.26
9	BAK1102	f	29	17	OS	23.44	281.2	163591	162999	12931	12884	2.25	2.69	-814	46	6941	12725	1.34	2.28
				18	OD	23.54	283.1	168569	167676	13507	13436	2.18	2.67	-794	48	7164	11703	1.34	1.36
10	BAK1067	m	14	19	OS	23.21	274.5	167548	166323	12625	12533	2.26	2.65	-825	45	6571	10293	1.12	1.42
				20	OD	23.2	274.1	165085	163366	12403	12274	2.26	2.66	-819	49	6543	11146	1.03	0.8
11	BAK1005	f	30	21	OS	24.79	300.7	167534	166694	15149	15073	2.28	2.63	-797	47	7727	10905	1.08	1.05
				22	OD	24.77	300.1	167421	164302	15078	14797	2.29	2.6	-766	52	7737	11887	1.08	1.51
12	BAK1090	f	27	23	OS	22.78	269.4	172300	170908	12505	12404	2.1	2.58	-920	47	6644	10086	1.18	0.85
				24	OD	22.87	270.3	170461	168683	12454	12324	2.21	2.61	-913	48	6556	10762	1.26	1.42
13	BAK1021	f	23	25	OS	22.28	263.4	176261	172432	12229	11963	2.12	2.74	-738	58	6755	12876	1.16	1.38
				26	OD	22.41	264.5	176908	176289	12377	12333	1.99	2.57	-813	50	6715	11136	1.1	1.09
14	BAK1086	f	21	27	OS	22.61	266.2	173692	172474	12308	12222	2.16	2.47	-826	46	6576	10465	1.28	2.03
				28	OD	23.13	274.9	157550	155470	11906	11749	2.36	2.73	-710	41	6273	10001	1.27	2.61
15	BAK1034	f	24	29	OS	22.7	270.2	174855	172877	12766	12621	2.13	2.56	-901	47	6748	11895	1.37	1.6
				30	OD	22.59	268.5	175128	172232	12625	12417	2.15	2.68	-882	48	6729	11791	1.24	1.38
16	BAK8022	f	26	31	OS	23.08	275.3	176795	174125	13399	13197	2.12	2.69	-770	62	7572	16141	1.3	1.82
				32	OD	23.18	276.4	178006	174133	13599	13303	2.1	2.61	-747	63	7656	16573	1.26	1.51
17	BAK1064	f	20	33	OS	22.93	270.1	179804	174815	13117	12753	1.98	2.53	-772	58	7295	15275	1.15	1.38
				34	OD	22.85	269.4	185045	180876	13430	13127	2.03	2.55	-789	57	7447	14841	1.18	0.93
18	BAK8018	f	29	35	OS	25.06	301.6	177896	175852	16182	15996	2	2.46	-809	36	8167	8977	1.07	1.27
				36	OD	25.12	302.6	189122	185655	17317	17000	1.96	2.59	-919	39	8501	9085	1.14	1.62
19	BAK1087	m	18	37	OS	24.67	294.2	183569	180115	15889	15590	2.14	2.67	-1102	46	7580	9012	1.23	2.09
				38	OD	24.82	296.1	180141	176405	15794	15466	1.98	2.68	-1103	47	7537	9857	1.23	1.73
20	BAK1091	f	23	39	OS	23.97	287.6	182809	181705	15121	15030	2.05	2.47	-1020	46	7578	9733	1.04	0.91
				40	OD	24.42	295.4	179526	179133	15666	15631	2.21	2.58	-1036	49	7727	9813	1	1.76

21	BAK8044	f	29	41	OS	23.85	284.5	187845	187186	15204	15151	2.11	2.44	-950	56	7858	12588	1.24	1.62
				42	OD	23.75	282.6	191326	187879	15280	15005	1.82	2.46	-902	55	7832	11950	1.18	1.78
22	BAK8001	f	33	43	OS	22.62	266.9	195648	190903	13934	13596	2.03	2.52	-1011	51	7358	11067	1.15	2.06
				44	OD	22.66	267.9	195119	193382	14008	13883	2.1	2.52	-1034	50	7480	11649	1.08	1.49
23	BAK1041	f	23	45	OS	23.17	273.4	195484	192492	14612	14388	1.92	2.55	-1123	50	7340	10075	1.12	1.11
				46	OD	23.09	273.4	195560	190206	14618	14217	1.98	2.55	-1179	51	7255	10306	1.16	1.02
24	BAK1040	f	30	47	OS	23.39	278.3	204356	192698	15828	14925	1.8	2.43	-992	67	8301	15546	1.12	1.73
				48	OD	23.64	282.8	197581	192354	15802	15384	1.93	2.49	-1050	56	8116	12763	1.09	3.4
25	BAK1012	m	22	49	OS	24.17	287	199409	193101	16425	15906	1.91	2.36	-1088	57	8110	12044	1.28	1.54
				50	OD	24.21	287.3	213260	205144	17603	16933	1.85	2.36	-1209	53	8359	10166	1.27	1.87
26	BAK1069	m	12	51	OS	22.99	269.5	205543	198468	14929	14415	1.89	2.45	-1191	38	7226	7252	1.22	1.82
				52	OD	23	269.9	203374	200949	14815	14638	1.92	2.29	-1167	32	7236	6448	1.13	0.89
27	BAK9019	m	44	53	OS	23.57	285.6	204434	200453	16675	16350	1.97	2.37	-1225	47	8135	9542	1.44	1.73
				54	OD	23.68	285	202701	197180	16464	16016	1.83	2.44	-1008	49	8161	9587	1.33	1.42
28	BAK1008	f	26	55	OS	22.68	266.6	211471	205166	15030	14582	1.96	2.34	-996	56	8158	13920	1.12	0.69
				56	OD	22.53	264.7	216764	210634	15188	14758	1.91	2.41	-975	54	8213	12937	1.07	1.38
29	BAK1070	m	10	57	OS	22.88	269.5	212748	210271	15452	15272	1.72	2.43	-994	39	7989	8927	1.08	1.26
				58	OD	22.81	268.6	209378	206225	15106	14878	1.95	2.34	-938	41	7815	9463	1.13	1.11
30	BAK1093	f	25	59	OS	23.28	274.6	221058	216209	16669	16303	1.95	2.41	-1098	53	8839	12306	1.34	2.34
				60	OD	23.42	277	219132	215681	16814	16549	1.91	2.38	-1088	44	8539	8922	1.28	1.24
